# “It Matters Who Defines It”—Defining Nutrition through American Indian, Alaska Native, and Native Hawaiian Worldviews

**DOI:** 10.1016/j.cdnut.2024.104429

**Published:** 2024-07-25

**Authors:** Tara L. Maudrie, Kaylee R. Clyma, Cassandra J. Nguyen, Victoria M. O’Keefe, Martin Reinhardt, Valerie Segrest, Melissa E. Lewis, Toni Stanger-McLaughlin, Nicole Redvers, Phoebe Young, Hope Flanagan, Electa L. Hare-RedCorn, Elsie M. Dubray, Alanna Norris, Kaylena E. Bray, Valarie Blue Bird Jernigan

**Affiliations:** 1 Department of International Health, Center for Indigenous Health, Bloomberg School of Public Health, Johns Hopkins University, Baltimore, MD, United States; 2 Center for Indigenous Health Research and Policy, Oklahoma State University Center for Health Sciences, Tulsa, OK, United States; 3 Department of Nutrition, University of California, Davis, CA, United States; 4 Center for Native American Studies, Northern Michigan University, Marquette, MI, United States; 5 Muckleshoot Tribal Member, WA, United States; 6 Department of Family and Community Medicine, University of Missouri School of Medicine, Columbia, MS, United States; 7 Native American Agriculture Fund, Citizen of the Colville Confederated Tribes, Spokane, WA, United States; 8 Department of Epidemiology and Biostatistics, Schulich School of Medicine & Dentistry, University of Western Ontario, Ontario, Canada; 9 Department of American Studies, University of Minnesota, Minneapolis, MN, United States; 10 Dream of Wild Health, Hugo, MN, United States; 11 Pawnee Nation Tribal Member, Pawnee, OK, United States; 12 Community Health and Prevention Research, Stanford University School of Medicine, Palo Alto, CA, United States

**Keywords:** American Indian, Alaska Native, Native Hawaiian, Indigenous, nutrition, qualitative, Indigenous research methods, nourishment, strengths-based

## Abstract

**Background:**

Adequately assessing nutrition is deeply important for understanding and addressing health inequities, especially for American Indian, Alaska Native, and Native Hawaiian (AI/AN/NH) peoples who have been subjected to the malnourishing effects of settler colonialism. Mainstream approaches to nutrition rely heavily on indicators of physical diet consumption, while ignoring the contributions of food to spiritual, social, and emotional health. Considering only physical aspects of dietary consumption paints a narrow picture of AI/AN/NH foodways and nutritional health further contributing to deficit-based narratives about AI/AN/NH peoples. To adequately understand and address the nutritional health of AI/AN/NHs, strengths-based approaches to nutrition that are rooted in Indigenous ways of knowing are urgently needed.

**Objective:**

The objective of this study was to define nutrition through AI/AN/NH worldviews.

**Methods:**

Three focus group discussions (*n* = 3) and in-depth interviews (*n* = 6) were conducted with 26 purposively identified AI/AN/NH food sovereignty experts and knowledge holders. Data collection and analysis were conducted iteratively. Through a process of open, focused, and axial coding, primary themes and supporting subthemes were established and refined, and a conceptual framework (the Indigenous Nourishment Framework) was developed to reflect the relationships between primary themes, subthemes, and the overall meaning of nourishment for AI/AN/NHs.

**Results:**

The Indigenous Nourishment Model contains 4 main domains of nourishment (physical, spiritual, emotional, and relational), and 8 subthemes representing concepts, practices, and beliefs, which contribute to overall nourishment.

**Conclusions:**

Despite the importance of physical aspects of nutrition, our findings assert that spiritual, emotional, and relational well-being are inseparable and integral to the overall goal of nourishment for AI/AN/NHs. The resulting Indigenous Nourishment Framework developed with a diverse group of AI/AN/NH food knowledge holders reflects the strengths of AI/AN/NH foodways and builds upon existing approaches to nutrition by offering new directions for approaching and measuring nutrition.

## Introduction

Nutrition is a key determinant of health throughout the lifespan [1]. In fact, inadequate nutrition is linked to increased risk of chronic diseases (e.g., type 2 diabetes and cardiovascular disease) and poor mental health in the form of depression and anxiety disorders [[2], [3], [4]]. As such, understanding and addressing nutrition is deeply important, especially for underserved populations, such as American Indian, Alaska Native, and Native Hawaiian (AI/AN/NH) peoples who experience significant health inequities [5].

Public health broadly considers nutrition at the household, community, and population levels, most commonly through indicators of individual dietary consumption [6]. However, these indicators of nutrition are strongly influenced by western medical philosophies, which suggest that food can be stripped of its emotional and social value to provide the exact amounts of nutrients required to maintain health [7,8]. Mainstream assessments of nutrition focus almost entirely on the nutritional value of consumed foods, and whether individual dietary consumption aligns with national dietary standards [[9], [10], [11]]. This overly simplified and narrow view of nutrition [11] overlooks the rich emotional and social interactions that occur through food production, preparation, and even the act of eating [10]. On the basis of standard nutrition assessments, AI/AN/NH peoples are generally considered to have poor nutrition. Narratives constructed from mainstream indicators of nutrition often position AI/AN/NHs as “vulnerable” and “at risk” because of dietary deficiencies, excesses, high rates of food insecurity, and food apartheid [[12], [13], [14], [15], [16]].

Although diet quality undoubtedly plays a significant role in health inequities faced by AI/AN/NHs, overemphasizing one aspect of nutrition (dietary consumption or physical nutrition) paints an incomplete picture of Indigenous nutrition. AI/AN/NH food practices (e.g., hunting, fishing, foraging, cultivating, processing, preparing, and sharing foods) are important not only for the foods they provide but also for the social and land-based relationships they create and strengthen [17]. Food holds deep emotional and spiritual importance to AI/AN/NHs and serves as an important medium for intergenerational knowledge transmission [18,19]. Furthermore, western approaches to nutrition frequently disregard Indigenous knowledge and worldviews, creating epistemic injustices, or more simply put, injustices of knowledge. Although AI/AN/NHs have lived in relationship with and cared for the only sustainable food systems North America has ever known, Indigenous knowledges and place-based knowledges continue to be dismissed as anecdotal and nonscientific. Despite continued calls for strength-based research and the efficacy of asset-based approaches to health, the worldviews and knowledges of AI/AN/NHs are rarely considered, measured, or uplifted as strengths in addressing nutrition [12,[20], [21], [22]]. To adequately address nutrition-related health inequities and to counter deficit-based narratives of AI/AN/NHs, strength-based conceptions and measures of nutrition grounded in Indigenous worldviews are critically needed [21]. Several factors underscore this point.

First, deficit-based nutrition research contributes to narratives that AI/AN/NHs face disproportionate health inequities (e.g., type 2 diabetes) because of individual dietary choices, “inferior” genetics, and body size, rather than the profound impacts of ongoing settler colonialism [12,13,[23], [24], [25], [26]]. Second, although Indigenous foodways are incredibly resilient and contribute to the continued thriving of AI/AN/NH peoples, many health promoting aspects of Indigenous foodways are not considered, measured, or uplifted as facilitators of good nutrition. Indeed, from AI/AN/NH perspectives, food is inseparable from spirituality, teachings, seasonal practices, and the continued thriving of Indigenous peoples and their ecosystems [18,27]. In the mainstream, subsistence practices, such as hunting, fishing, gardening, and foraging are recognized as ways to access healthy, nutritious foods. But for AI/AN/NHs, the benefits of subsistence practices transcend physical access to food, and by participating in land-based food activities, AI/AN/NHs cultivate profound connections to land, water, and to one another through cultural and social bonding [28,29]. Third, the intentional disruption of Indigenous foodways by settlers (e.g., slaughter of buffalo and burning of crops) has resulted in enduring traumas, which can only be repaired by restoring and revitalizing Indigenous foodways through culturally grounded approaches [27,[30], [31], [32]]. Alongside the more physical and tangible traumas inflicted upon Indigenous food systems, historical and contemporary epistemic injustices within the field of nutrition contribute to a structural oppression through ongoing settler colonialism. Defining nutrition through AI/AN/NH worldviews offers opportunities to restore and strengthen the reciprocity-based relationships inherent in AI/AN/NH foodways, and enhance mainstream approaches to nutrition by emphasizing the important role of nutrition in holistic well-being, not just for individuals, but for communities and entire ecosystems. Lastly, defining nutrition through AI/AN/NH worldviews provides a way to correct epistemic injustices by considering important aspects of nutrition beyond dietary consumption [33].

Despite the importance of understanding and measuring physical nutrition to address health inequities in AI/AN/NH communities, we assert that approaches to nutrition must consider the holistic nature, worldviews, and beauty of Indigenous foodways. Therefore, the purpose of this study was to engage AI/AN/NH food sovereignty experts and knowledge holders to define nutrition-related concepts through their worldviews and values.

## Methods

### Study design

Previous community-based participatory research (CBPR) with AI/AN/NHs from the current research team (TM and VBBJ) found that mainstream definitions of food security do not align with the worldviews and values of AI/AN/NHs. In response to these concerns, as part of a larger CBPR project with AI peoples (Grant #: 5R01MD011266-05; primary investigator: VBBJ), we endeavored to understand what food security means to AI/AN/NH peoples. Our study was informed by Grounded Theory and Indigenous Research methodologies, as well as our relational research epistemology (which we elaborate on later), guiding our selection of data collection and analysis methods [34,35]. The study utilized focus group discussions (FGDs) and in-depth interviews (IDIs) with purposively recruited AI/AN/NH food sovereignty experts, knowledge holders, and researchers. Indigenous research methods were utilized through which project participants were able to opt into coauthorship on resulting dissemination materials and to having their quotes and ideas attributed to them by name [36,37]. The study received institutional review board approval from Oklahoma State University Center for Health Sciences (IRB#: 2019012) and written consent was obtained virtually via REDCap, a secure web-based platform designed to capture data for research studies hosted at Oklahoma State University Center for Health Sciences [38].

In order to gather detailed perspectives and nuanced dialog, AI/AN/NH food sovereignty experts and knowledge holders were purposively recruited via email [39]. Participants were identified through the research team’s knowledge and connections within the field of AI/AN/NH nutrition and food sovereignty, and through brief literature reviews that identified relevant authors. Participants represented a range of positionalities through their tribal affiliations, geographic locations (e.g., urban, rural, reservation, and suburban), their roles in food sovereignty initiatives, national food security programs, community education, food production, and as cultural leaders of their respective communities (e.g., elders, ceremonial leaders, and academics).

This study utilized FGDs and IDIs with purposively recruited AI/AN/NH food sovereignty experts, knowledge holders, and researchers. The facilitation guides for IDIs and FGDs (see Supplemental Materials) were developed through iterative meetings with the study implementation team (TLM, KC, and VBBJ) and were informed by the team’s previous experience conducting food security research within Indigenous communities. In August and September 2022, three virtual FGDs (*n* = 3) were conducted with a total of 20 participants. To accommodate participants who were unable to attend FGDs, additional virtual IDIs (*n* = 6) were conducted between September 2022 and December 2022. All FGDs and IDIs were facilitated by TLM, and KC assisted in technical facilitation for FGDs. The first two focus groups consisted of 8 participants in each, whereas the last FGD had 4 participants. The length of FGDs ranged from 81 to 91 min (mean 85 min). The 6 IDIs ranged in length from 37 to 77 min (mean 53 min). Participants were provided with a $50 visa gift card as a token of appreciation for their time.

FGDs and IDIs recorded and transcribed the audio verbatim. TLM composed analytic memos following each FGD, IDI, and during data analysis to capture emerging themes and facilitation considerations for ongoing FGDs and IDIs. Using Atlas.ti Mac (version 23.1.1) to facilitate analysis, TLM iteratively analyzed transcripts alongside data collection. To aid us in our goal of developing a culturally grounded conceptualization of nutrition, we utilized a grounded theory method for analysis [34]. The grounded theory method consists of a systematic approach to coding with the ultimate goal of developing theory grounded in the participant experiences [34]. Our grounded theory method for analyses consisted of three main stages: open coding, axial coding, and selective coding [34]. TLM and KC independently open-coded the first two FGD transcripts and met to compare inductive codes, coding approaches, and develop an initial codebook. TLM coded the remaining transcripts iteratively along with data collection, adjusting the codebook through meetings with the study team (KC and VBBJ) to accommodate emerging insights from ongoing interviews. In the final interviews, thematic saturation was achieved, with no new themes emerging from the last two IDIs. Once all transcripts had been coded, TLM engaged in axial coding, grouping codes into similar topics or ideas, before engaging in selective coding to establish qualitative themes [34]. Themes were refined through meetings with the study team (KC and VBBJ) and TLM’s academic advisor (VMO). To reflect the complex relationships between the aspects of nutrition, TLM developed a conceptual framework that was refined through meetings with the study team (KC and VBBJ), members of her dissertation advisory committee, and through written feedback from project participants who opted into coauthorship in this article via the consent form as described earlier. Despite the vast heterogeneity of AI/AN/NH communities, the qualitative sample reflected this diversity and included a range of perspectives. Many of the insights gained through FGDs and IDIs shared similarities across cultural groups, indicating that we may have also achieved theoretical saturation [40].

### Epistemology

Earlier, we discussed the epistemic or knowledge injustices that are created and perpetuated when Indigenous knowledge is disregarded in nutrition and research, which invites even deeper questions into the field of epistemology, or more simply put the science of knowledge. Before presenting our results, we examine the epistemologies that inform this research, and other indicators of qualitative research trustworthiness, enabling transparency for the reader to better understand and draw their own conclusions about our research approach.

Traditionally, qualitative research operates under a constructivist epistemology. A constructivist epistemology asserts that there are multiple realities and that those realities are subjective and socially constructed [[41], [42], [43]]. More simply put, the way in which people understand the world, construct their realities, and interpret their experiences is individual [[41], [42], [43]]. A constructivist epistemology is in stark contrast to an objectivist epistemology often employed by quantitative research, which assumes that there is one truth and reality, and our goal as researchers is to measure this objective truth by minimizing potential biases as much as possible [44,45]. A relational (or Anishinaabe) worldview is similar to a constructivist epistemology in that it recognizes that there are multiple realities and is contextually grounded [46]. A relational epistemology acknowledges that a person’s reality consists of relationships they hold with themselves (including mind, body, and spirit), their families, their communities, plants, animals, land, water, and their ancestors (and more) [47,48]. However, fundamental differences exist between relational and constructivist worldviews. Relationality considers types of knowledge that may not be included in a constructivist epistemology [49]. Examples included incorporating values derived from the interaction of emotion and experience including teachings, ceremonies, stories, songs, and ecological knowledge [[47], [48], [49]]. Many Indigenous teachings, stories, and ceremonies have protocols to protect sacred knowledge and some types of knowledge may not be shared outside of community or ceremonial contexts [[49], [50]]. Nevertheless, the values that underlie these teachings inform our experience and research as Indigenous peoples. The constructivist epistemology (and much of qualitative research through the concept of transferability) is interested in generating theories or explanations that apply outside of the specific context in which the research was done [[51], [52], [53]]. A relational epistemology is inherently place based and does not need to apply to other contexts to have value; its value is derived from its ability to fulfill a role in a relationship, not from its ability to apply to other contexts [35]. Indigenous teachings guide our experience and research, informing our relational epistemology, which underpinned our study design, data collection methods, and analyses.

### Relationality

As part of our Indigenous research practice, as well as qualitative research transparency and reflexivity, we offer the following context and relationality statements from the study implementation team. Although reflexivity in qualitative research is frequently practiced as a way to separate the experiences of the research team from their research processes [54,55], we want to emphasize that by offering these relationality statements we do not seek to separate our experiences and positionalities from our research. Instead, we are offering our own relationality statements to share who we are, how we come to this work, and how this connectedness is fundamental to understanding this research. Tara L. Maudrie (TLM) is a citizen of the Sault Ste Marie Nation of Chippewa Indians and has spent most of her life as an urban Native person. Her personal motivation for engaging in Indigenous food systems research is fed by her personal and familial connections to food sovereignty, through hunting and fishing, as well as her academic training in exercise science, nutrition, and social behavioral sciences. Her analyses, interpretation of findings, and the development of the Indigenous Nourishment Model are informed as much by her cultural values and teachings, as they are her training in qualitative and Indigenous research methodologies. Kaylee R. Clyma (KRC) is an enrolled citizen of the Cherokee Nation and a research project coordinator. She has experience in implementing food sovereignty initiatives using CBPR in partnership with Indigenous communities. Valarie Blue Bird Jernigan (VBBJ) is an enrolled citizen of the Choctaw Nation of Oklahoma, a Professor of Medicine, and an intervention scientist with Indigenous communities. She has lived experience in traditional foods and foodways as well as scientific expertise in food sovereignty, Indigenous health, and nutrition.

## Results

In Methods section, we described our epistemology and relationality as researchers, both of which call for a slightly different approach for presenting our results than that is typical for a mainstream qualitative article. In line with Indigenous ways of knowing, the Indigenous knowledge holders who participated in our research were able to opt into having their quotes attributed to them by name. Below we present the main themes of our research supported directly by participant quotes and provide our interpretation of these themes as a collaborative authorship team that includes both participant and researcher perspectives.

Although the project intended to inquire into the specific topic of food security, we adapted to the priorities expressed by participants demonstrating the iteration and flexibility needed in qualitative research. Therefore, this article explores nutrition more broadly, rather than focusing solely on food security. Before presenting the developed conceptual model, we present two major themes. First, participants expressed frustration with dominant societal conceptualizations of nutrition and passionately shared the importance of centering AI/AN/NH worldviews in nutrition for all people. Second, participants described the importance of understanding nutrition holistically.

### Priorities of Indigenous nutrition and the importance of defining nutrition through Indigenous worldviews

Many participants remarked on the importance of defining nutrition through their Indigenous worldviews. Martin Reinhardt shared, “It really does matter who controls access to food. It matters who defines it. It matters that we are able to speak on behalf of the plants and animals and continue to speak on behalf of them and protect them, because that’s our responsibility... We don’t want food equity. I don’t want to eat the same stuff that the rest of the Americans are eating. That’s not what I want. I want to have what we call in Anishinaabe, mino bimaadiziwin [living the good life; living the life Creator intended for us].” Similarly, Phoebe Young stated, “We want to be able to define what healthy looks like to us, and what nutrition looks like to us, and what’s a dignified way to get our food…How are we assigning shame and blame and dignity to the different ways that people get food? How does that affect our relationship to different food systems?”

Kamuela Enos describes the challenges in translating between Indigenous and western epistemologies by saying, “The reason I like to think in our Native language first is because you can’t casually use words between languages because they come from different epistemologies, and that when we try to fit into contemporary terms, we’re trying to fit our language into the construct of English. English is great, but I really believe that [ancestral] language was taught to you by the land to learn how to survive within it…For us, the word ʻto eatʼ and ʻto growʼ are the same thing.” Kamuela went on to explain his frustration with dominant knowledge systems and his hope that Indigenous knowledge systems will be recognized for the value they hold for all people. He elaborated saying, “I’m tired of Native people having to justify our practices to the dominant system…I like to be expansive in thinking, and I think, tell the system that, hey, we have thousands of years of R&D [research and development] embedded in the practices that are in this place, and that we have community members that developing cultural competencies and contemporary fluencies in the system, and that you’re invited to learn about these practices with us because not just to us, but they’re important to all of you to learn.”

Several participants summarized the holistic importance of nutrition to AI/AN/NHs. Electa Hare-RedCorn stated, “Food is not only medicine, food is our spirituality, and that brings us back to how we’re related to one another.” Valerie Segrest expanded specifically on the concept of food security by saying, “Food security is, yes, on the physical level, good nutrition and promoting longevity in life, but it’s also part of keeping that stitch together in our social fabric, and that is what maintains our social-emotional health as well, which is equally important now more than ever, really.”

### Defining nourishment through Indigenous worldviews

**FIGURE 1:**
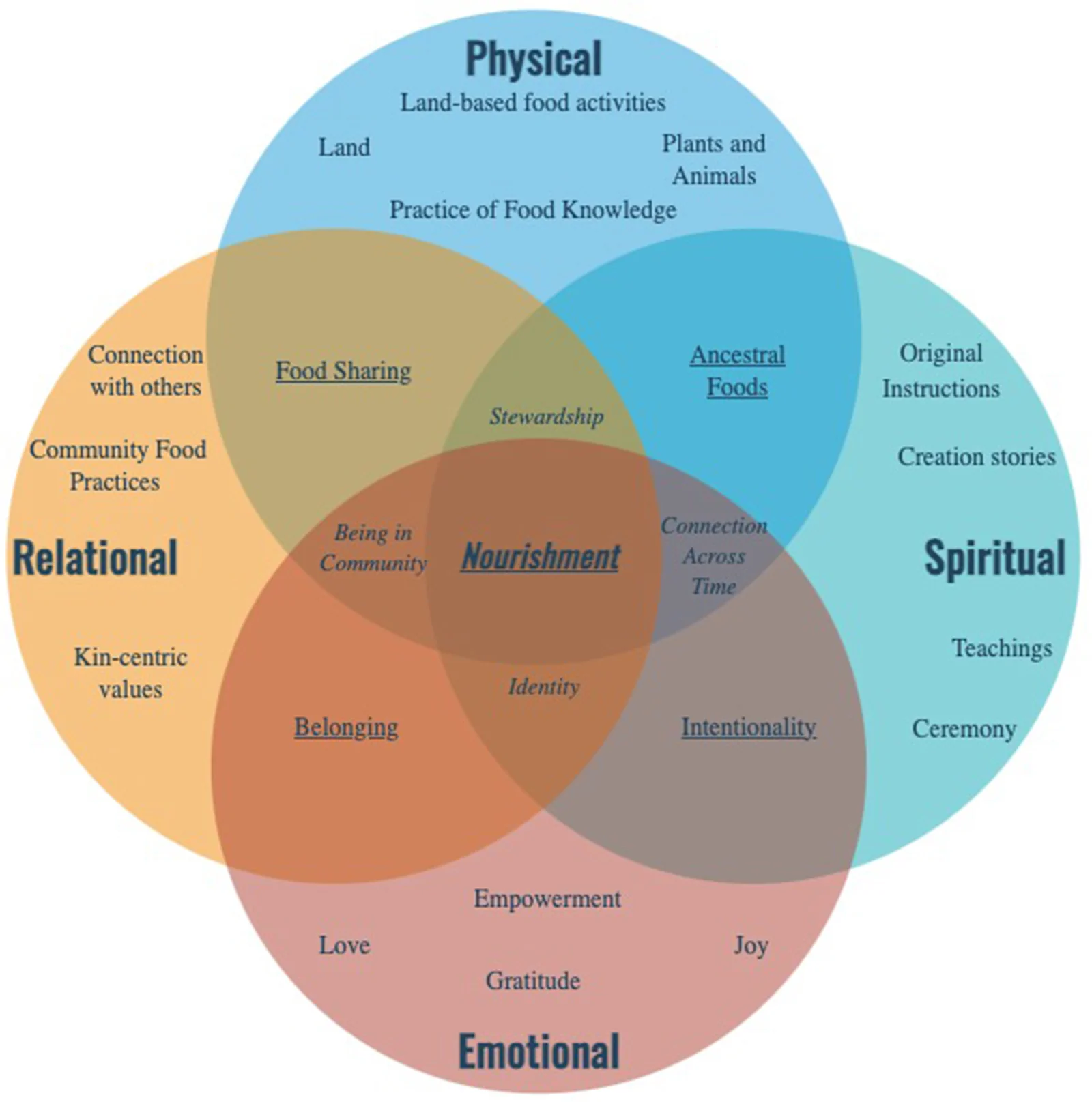
The Indigenous Nourishment Model.

Beyond the two themes shared above (AI/AN/NH nutritional priorities, and importance of defining nutrition through AI/AN/NH worldviews) qualitative analysis revealed 4 main domains composing overall nourishment: physical, spiritual, emotional, and relational. However, many themes did not fit neatly in these 4 domains and there was overlap with aspects of multiple domains. The overlap between themes and domains aligns with AI/AN/NH worldviews that often emphasize the interconnectedness of the world [56]. To represent the relationships and overlap between themes and domains, a conceptual framework of Indigenous nourishment was developed (Figure 1).

### Four main domains of nourishment (physical, spiritual, emotional, and relational)

#### Physical

In the mainstream nutrition is discussed in terms of physical health processes, such as the consumption, metabolism, utilization, and excretion of nutrients [1]. Participants expressed that food practices, such as gardening or preparing foods, are as much a part of physical nutrition as actually consuming foods. A female participant shared, “By taking care of the seeds, tending to garden, really having that close relationship, my hands really connected to my food at all the different stages, really has helped me have what I think is a healthier relationship with food. And I don’t mean just nutritionally but on a lot of different levels.” Another anonymous female participant discussed the importance of physical food practices for connecting AI/AN/NH peoples to their overall health. She explained, “I’ve seen a lot of Native people that literally start working with their meat, start learning about whitefish, and then it’s something that opens them up and activates them in ways to where they’re open to learning about food more, in general, because there’s these traditional ties…What are the nutritional values of bison? Why are white fish and bison some of the most healthy things that anybody could ever eat? And how is that related to us as Native people? …I’ve just seen people that aren’t really interested in food a certain way or even their diet, if they start learning about their ties to Indigenous foods or just practicing their Indigenous food sovereignty…that’s something that does coincide with them wanting to learn more about how to be healthy in general.” This domain communicates the importance of engaging in physical food practices, such as hunting, fishing, gardening, gathering, food preparation, and cooking, for cultivating healthy relationships with foods.

#### Spiritual

Spiritual aspects of nutrition, including the role of food in ceremony, creation stories, cultural teachings and fulfilling Original Instructions (guidelines for living in good relationship with all of creation), were frequently discussed among participants [57]. It is important to note that spirituality encompasses many aspects of Indigenous well-being and worldviews. In many Indigenous communities, aspects of physical, emotional, and relational well-being are manifestations of spirituality, meaning that the spirituality permeates every aspect of being, including food and nutrition. Toni Stanger-McLaughlin shared, “Every [Native] community I go to, their creation story is somehow tied to food…It’s usually always reciprocal in that something is sacrificing itself for the people to survive and then in return, the people will take care of those ecosystems, the plants, the animals, the air, the water, and then they will also one day return to the land to feed those systems once again.” Another participant, Anthony Barela, illustrated the importance of fulfilling sacred responsibilities through food by stating, “If some of our Original Instructions are to care for one another and to allow for one another to survive, then that must mean that we also allow one another to eat, to have food. So, I feel like it’s just embedded. To our instructions for how we care for one another is to provide food for one another.”

#### Emotional

The role of emotion in food was apparent, as many participants described how their traditional foods and food practices counteracted negative emotions and kindled positive emotions. Phoebe Young described the complex emotions associated with food by saying, “There’s, I think, a level of shame that we attached to certain types of food and how people eat. I think that one of the really big components of restoring healthy relationships to food is taking that shame away and really recognizing that there’s a lot of love and empowerment that can go into the relationship that you have with your food…I think that that gratitude is what fills up the holes that shame tries to fill up.” An anonymous female participant shared, “There’s also a joy that comes with being able to participate in cultural food systems. Even if you're not currently on your homelands or with other people from your Tribe, you can still eat those foods, and there’s still that connection that there wouldn’t be in that.”

#### Relational

Lastly, participants outlined the relational nourishment felt from connection with other humans, plants, animals, water, and land. Illustrating this sentiment, Nicole Redvers shared, “You can create relationship to food, no matter where you are, if you’re a guest or a visitor on a land, that relationship can be incredibly powerful to the same extent of eating your own foods that you might not even have connection to.” Expanding on relational connection within foodways Kateri Tuttle said, “I think it does come down to kinship systems, recognizing that we are part of this system or homelands. We evolved along with all the plants and animals to be a part of those systems and none of us can function without the others.” As described by participants, food facilitates and strengthens relational connection through the recognition of the interconnectedness and interdependence of humans, plants, animals, and lands and aspects of their foodways.

### Intersections of two domains of nourishment

#### Ancestral foods (physical and spiritual)

Although ancestral foods are often praised for their nutrient profiles (e.g., low fat and high fiber) and their contributions to physical nourishment, participants frequently remarked on the importance of ancestral foods for both physical and spiritual well-being. Hope Flanagan described advice she received saying, “I know those elder ladies when I was young, they’d always say, eat those wild foods because that’s the spirit you’ll have. You take in the life of whatever you eat. So, I was always encouraged to eat wild rice, whatever you could hunt or trap, whatever you could catch to eat, whatever berries that you gathered. Always try to eat as much as you can from the wild foods.” Another participant, Gary Ferguson, pointed out the shortcoming of western nutrition approaches in recognizing the spiritual aspects of food by sharing, “This more western based approach toward traditional foods has caused us severe problems related to our own way that we look at our food system which is broken down into biochemical properties…When you think of food from a cultural perspective, it’s way more than just those biochemical properties. It’s a connection to the spirit of the food, the animals, the plants.” In addition to the physical nutrition provided by traditional/ancestral foods, these foods sustain spiritual connections.

#### Intentionality (spiritual and emotional)

Participants identified intentionality, or being deliberate in their thoughts, hopes, beliefs, and desires about food, as an important spiritual and emotional practice that facilitates connection to food. Phoebe Young shares, “Having access to and learning more about our traditional foods, the history behind them, how we take care of them, language behind them, stories behind them, I think helped me look a lot more toward food as more as a gift and more as something that you shouldn't be ashamed [about]. That you need [food] to show gratitude toward and share with others and that you need to live.” Ruminating on the deeper meaning of foods, including understanding the cultural and historical significance of foods within culture facilitates positive spiritual and emotional well-being.

#### Belonging (emotional and relational)

As expressed by participants, food plays an important role in connecting us to our relations and to an emotional sense of well-being, resulting in an overall sense of belonging. Valerie Segrest shares, “When we are part of our food system and an active participant, we feel that we belong to something, be it the food that we're tending to or advocating for or the people that we are feeding, and that sense of belonging is so critical to maintaining our self-worth and our ability to be generous in the world and be an active participant of reciprocity.”

#### Food sharing (relational and physical)

Sharing foods is an important practice in many AI/AN/NH communities that strengthens relational bonds, while providing physical nutrition through shared foods. An anonymous female participant said, “With so many Native folks, we don’t go somewhere without bringing a gift, and that gift is almost always food…It’s an important way to express gratitude, and it’s an important way to establish or re-establish your support and existing relationships.” She went on to say, “It’s [sharing food] an important ritual that reinforces relationships and security and sense of place within the community that I think is really important. Also when you’re sharing food, there’s this constant … trading of knowledge as well with that that's kind of can break the ice if you’re in a place where you don’t know all the people, or you always take away different tips for how people are doing it…That reinforces this kind of constant learning for place-based knowledge that’s really important to those food resources and also to us as salmon people…But it’s never just about the food, it’s about the conversations around the environment in which that food is procured, that’s also an important ritual in that sharing I think.” Sharing foods not only contributes to physical nutrition, but also to building and reinforcing relational connections through the sharing of knowledge and resources.

### Intersections of three domains of nourishment

#### Connection across time (physical, spiritual, and emotional)

Food facilitates connection across time through the physical consumption of ancestral foods, and the spiritual and emotional significance of those foods across generations. Phoebe Young discussed this concept, noting that her ideas of ancestral memory are strongly influenced by Ho-Chunk Chef Elena Terry. Phoebe shared, “The feeling that you get when you eat your foods that your ancestors ate, that’s that ancestral memory… when you smell it and when you prepare it, and you hold that food in your hand and it feels like a relative…You can connect with your ancestors in the past, and in the present, and in the future …I hope that this is the food that guides my ancestors and my children home because it was for me.” Many participants noted the power of foods to connect AI/AN/NHs across the past, present, and future, suggesting that for some eating ancestral foods is an act that transcends time, connects them to the past, present, and future of their traditional foods and their culture, and creates positive emotions.

#### Cultural identity (spiritual, emotional, and relational)

Participants emphasized the role of food in cultural identity for AI/AN/NHs. Phoebe Young says, “We’re made up of all of these foods that came from the land that we came from. All of these plants and animals have all given their gifts so that we can live. A huge part of that has to do with food. I think that has to do… When you’re like, ‘I come from this particular place,’ that’s also saying, ‘I come from these food systems’…I might not have grown up my whole life eating walleye or wild rice, but I know that my family and my ancestors did… I think that that’s something that’s really nice and beautiful. Because that remains true, regardless of what connection you as an Indigenous person or as a Native person have to your home community, there’s always a story behind the food and the stories are how you describe the relationships that you have to the food that you eat and to what’s given you life. That’s true no matter where you are.” Participants emphasized that cultural food identity transcends geographic location and instead is a connection fostered from the recognition of spiritual, emotional, and relational connections to food.

#### Being in community (physical, emotional, and relational)

Many participants emphasized the importance of being in community for sharing foods, food practices, and food knowledge. An anonymous female participant shared, “There are certain things that can only be done together, with each other, in community and I feel with some of our food ways that really applies, there are only certain things like feasting. As an individual, you could feast something, but I think it’s more intended to be done as a group, even if it’s a small group and even practicing specific food ways, like processing foods, doing it in a good way, because some of those things are intended to be done in community.” Elsie M. DuBray elaborated on this by saying, “At the basis of what we’re talking about is that connection. It is more about its connection, its relationship, its being in community with each other, with whatever land, with whatever food, but especially our own. I’m constantly trying to figure out how to juggle those things, but it’s about the connection, it’s about the gratitude, it’s about the reciprocity. It’s about being in community with things.” As expressed by participants being in community is key to relational, physical, and emotional well-being through food.

#### Relational stewardship (relational, physical, and spiritual)

Relational stewardship through caring for food systems was discussed by many participants. Anthony Barela described the reciprocity established through stewardship by saying, “By fulfilling one of our duties in the way that we produce food for ourselves…We’re providing food for ourselves, for our community, and that extends beyond the human community. In acting in those ways of reciprocity, those other creatures also give to us in better ways…We provide more corn, apples, etc., to the deer. Maybe the deer are healthier. They have more children. More of the children survive winters and that leads to more deer for humans to eat. They offer more of themselves because we offer more of what we produce for them as well.” Essentially, stewardship of food systems involves physical practices that fulfill spiritual and relational responsibilities to care for all aspects of one’s food system.

## Discussion

Through engagement with AI/AN/NH food sovereignty experts and knowledge holders, we developed the Indigenous Nourishment Model, a culturally grounded model of Indigenous nourishment. This Indigenous Nourishment Model supports the resurgence of Indigenous ways of knowing and being in the field of nutrition, and provides considerations for healing and transcending the foodways trauma inflicted upon AI/AN/NH peoples [58]. Furthermore, this model responds to calls for strength-based and culturally grounded approaches to nutrition for AI/AN/NH communities, and offers new considerations for assessing public health nutrition [12,14,20].

The Indigenous Nourishment Model visually (Figure 1) accentuates the interrelatedness of physical, spiritual, emotional, and relational practices for overall nutrition. The Indigenous Nourishment Model aligns with other AI/AN/NH models of health and well-being that center connectedness and relationality as key determinants of well-being [17,56,59,60]. The holistic perspective on health communicated by the Indigenous Nourishment Model shares common ground with social determinants of health (e.g., education, socioeconomic status, and neighborhood food environment), which are commonly used to understand a range of factors that may influence nutritional and overall health. Although the social determinants of health offer a holistic approach to understanding health, they are primarily shaped by western and dominant society values. In contrast, the Indigenous Nourishment Model is rooted in Indigenous worldviews, providing novel considerations and perspectives on nutrition. Placing physical nutrition into perspective within the larger concept of Indigenous nourishment, provides communities, nutrition professionals, and researchers with direction for framing, developing, and evaluating food and health-related initiatives. The Indigenous Nourishment Model also provides a solution to ongoing epistemic injustices within the field of nutrition, and represents an important step toward indigenizing approaches to nutrition in clinical and community practice. More broadly the Indigenous Nourishment Model can be used by the field of nutrition for expanding our understanding of what it means to be nourished for all peoples.

Published nutrition research often acknowledges the importance, and even intersection, of nutrition in achieving physical and mental well-being [61]. As previously described, physical nutrition is undoubtedly important to maintaining health; however, many AI/AN/NHs assert that food is more than just physical outcomes of consumption and metabolism of nutrients [62]. Participants expanded upon this concept by describing the importance of physically connecting with food through cultivation and preparation of foods. Physically participating in foodways through gathering and processing foods provides physical activity and social opportunities, which may have an impact on holistic health beyond the nutrition the produced foods provide [19,63,64].

Although spiritual well-being is rarely explored or discussed in mainstream nutrition literature, research in AI/AN/NH communities demonstrates that spiritual well-being is deeply intertwined with all aspects of well-being [[65], [66], [67]]. As described by our participants and published literature in AI/AN/NH communities, food is inherently a part of spiritual practices, including ceremony, creation stories, and cultural teachings [18,68,69]. Beyond the role of food in important spiritual practices, foods themselves, especially traditional/ancestral foods, carry spirituality [18,70,71].

The role of emotion in eating habits has been explored in the mainstream literature, mostly in describing the role of emotions in dietary consumption. The majority of emotional nutrition research is often framed in a deficit discourse, positioning emotions as a cause of poor eating habits and a barrier to healthy eating [[72], [73], [74]]. However, some published research shows that foods can increase positive emotions, and even that positive emotions increase desire for healthy foods [75,76]. As described by our participants, food has the power to cultivate positive emotions, such as joy and gratitude and even to combat the effects of negative emotions such as shame.

The relational domain of nourishment represents participant perspectives on the importance of relationships created through food and fulfilling responsibilities to care for one another through food. Previous research with Indigenous communities has emphasized that revitalizing Indigenous foodways also restores and strengthens relationships with other humans and more than human aspects of our foodways (e.g., land, water, plants, and animals) [77,78]. Furthermore, mainstream research has documented the positive impacts of social connection on dietary quality, primarily through sharing in food cultivation, preparation, and meals with others [[79], [80], [81]].

One of the major strengths of this project is the potential utility of the Indigenous Model of Nourishment. The Indigenous Nourishment Model was developed in partnership with a diverse group of knowledge holders and reflects the strengths and beauty of Indigenous foodways. This model provides a potential framework for developing, implementing, and evaluating food-related initiatives within AI/AN/NH communities and beyond. One of the unique contributions of the project is the recognition of participants as knowledge holders and experts. Each participant brought their own lived experiences, cultural teachings, and professional expertise to the project. As one way to honor their unique positionalities and invaluable knowledge, all participants were invited to partner in the research project through opting into having their quotes attributed to them and through coauthorship of dissemination materials.

However, this research is not without limitations. The tension between creating culturally specific models, and models that have resonance across various cultures has been discussed by various Indigenous and allied scholars working in collaboration with Indigenous communities [82,83]. This project engaged participants across Turtle Island and therefore may have some cross-cultural validity or resonance, but aspects of the Indigenous Nourishment Model may not resonate with all AI/AN/NH communities. However, this model could be culturally adapted, or may provide a starting place for communities to create their own culturally specific models of nourishment. Throughout the project participants noted that although there were significant geographic and cultural differences among them, many AI/AN/NH peoples share common values and purposes related to food, well-being, and worldviews. It is important to note that this does not in any way mean that AI/AN/NHs are a monolith or share a monoculture, but rather that our cultural wisdoms transcend geography and connect us universally in our shared goal of nourishment. Our research sample consisted exclusively of AI/AN/NHs, which was crucial for achieving our goal of developing a model of nutrition that is reflective of AI/AN/NH worldviews. Although we consider this a strength of our research, some may consider the lack of representation of dominant society perspectives in our research a limitation. As previously discussed, mainstream discourse often perpetuates deficit-based narratives about AI/AN/NH nutrition, where our model highlights numerous strengths in how AI/AN/NHs conceptualize and approach nutrition. Further research is needed to critically examine whom these deficit-based narratives and dominant society’s definitions of nutrition serve and how AI/AN/NH conceptualizations of nutrition (and other health-related concepts) can challenge ongoing settler colonialism by addressing epistemic injustices. The theoretical framework we presented in this paper explores the interconnectedness and holistic nature of nutrition for AI/AN/NHs. However, further work is needed to operationalize this model and make actionable recommendations for using the Indigenous Nourishment Model in individual, household, community, practice, and policy contexts.

### Future directions

The model presented in this article provides direction for understanding and evaluating the holistic impact of nutrition and food sovereignty initiatives in AI/AN/NH communities. Although AI/AN/NH peoples have long understood and recognized the importance of holistically considering nutrition, more empirical research is needed to understand how nonphysical aspects of nutrition may play a role in nutritional health and overall well-being [60]. Although the Indigenous Nourishment Model developed in the current research reflects the perspectives of AI/AN/NHs, aspects of this model of nutrition likely will resonate with people of varying races, ethnicities, and backgrounds. Indeed, food is a strong reminder of how connected we are—and just how much we have to learn from one another. Our team is currently building upon this collective work to develop quantitative measures of nourishment on the basis of the Indigenous Nourishment Model. We hope that this model and the forthcoming measure can aid us in holistically understanding and addressing nutrition for all peoples.

## Author contributions

The authors’ responsibilities were as follows – TLM, KC, VBBJ: designed research, conducted research, analyzed research, and wrote the paper; CJN, VMO, MR, VS, MEL, TS-M, NR, PY, HF, EH-RC, EMD, AN, KEB: contributed to the interpretation of data and contributed to the writing of the manuscript; TLM: is responsible for the final content of the manuscript; and all authors have read and approved this manuscript for publication.

## Conflict of interest

The authors report no conflicts of interest.

## Data Availability

Data described in the manuscript, code book, and analytic code will not be made available in accordance with Indigenous data sovereignty practices.
