# Supplementary material for: “It Matters Who Defines It”—Defining Nutrition through American Indian, Alaska Native, and Native Hawaiian Worldviews
Source: Curr Dev Nutr. 2024 Jul 25;10(Suppl 2):104429. doi: 10.1016/j.cdnut.2024.104429 (PMC13615723; doi:10.1016/j.cdnut.2024.104429)
Supplement: Supplementary file 1 [file mmc1.docx]

**Supplementary Materials**

Note: The facilitation guides provided below were used to facilitate discussions, meaning not every single question was asked in each data collection event, and probes were used to follow the natural flow of conversation.

**Focus Group Discussion Facilitation Guide**

**Introduction to Focus Group**

*Thank you everyone, we are so glad you are here. As you know the topic today is on how food security is defined by AI/AN peoples. We recognize that there are likely varying levels of understanding and different interpretations of the term food security among those of us gathered today. Our intention today is to listen and learn from you. This project is already made so much stronger by your presence, no one can speak better as to what is happening in your communities than you can, we are here humbly seeking your expertise. We hope this will be an informative and energizing conversation. My goal as the facilitator is just to help the conversation move along, please feel free to respond to what others have said or even to ask your own questions as you see fit.*

*Before we get started with the focus group questions, we want to make space to introduce ourselves. We know that relationships are an important part of research and for some of you this may be the first time you are meeting. We will take a few moments to do introductions. You can introduce and identify yourself in whatever way feels best to you. I’ll start by introducing myself and then we’ll take turns passing introductions until everyone has introduced themselves.*

**Focus Group Discussion Questions**

- *Can you share what food security means to you?*
  - *What do you think food security meant to your ancestors?*
  - *To your community?*
- *What is it like to be food secure or food insecure?*
- *Are there aspects of food security that are specific to being AI/AN?*
  - *Are there inter-generational effects of food insecurity?*
  - *Are cultural or traditional foods related to food security?*
- *In recent years there has been scholarship to redirect discussions of food systems to discussions on what it means to be part of a relational and kin-centric foodway. Does food security mean something different in the context of a relational or kin-centric foodway?*
- *I wanted to share the USDA’s definition of food security. (*shares slide with the following definition*)*
  - Food security is defined as: “access by all people at all times to enough food for an active, healthy life."
    - Food security includes at a minimum:
      - The ready availability of nutritionally adequate and safe foods.
      - Assured ability to acquire acceptable foods in socially acceptable ways (that is, without resorting to emergency food supplies, scavenging, stealing, or other coping strategies).
  - *Can you share any reactions you have to this definition and what you would change, add, or dispute in this definition?*
- **Share slide with USDA Food Security Screener**
  - *How does this measure differ from how you think food security should be measured?*
- *Before we end today, is there anything anyone would like to add?*
  - *Are there any questions you wish I would have asked or topics you wish we had covered?*

**In-Depth Interview Facilitation Guide**

**Introduction to Interview**

*Thank you for being here, we are so glad to be talking with you today. As you know the topic today is on how food security is defined by AI/AN peoples. We recognize that there are likely varying levels of understanding and different interpretations of the term food security among those who are participating in this project. My intention today is to listen and learn from you and your experiences. This project is already made so much stronger by your presence, no one can speak better as to what is happening in your communities than you can, we are here humbly seeking your expertise. We hope this will be an informative and energizing conversation. My goal today is really to listen and learn from you, I have a few questions I would like to ask you today but we are hoping this will be conversational.*

*Before we get started with the interview questions, I want to make space to introduce ourselves. We know that relationships are an important part of research, and our community accountability structures. I’ll start by introducing myself and then I can pass to you to introduce yourself in whatever way feels best to you.*

**Interview Questions**

- *Can you share what food security means to you?*
  - *What do you think food security meant to your ancestors?*
  - *To your community?*
- *What is it like to be food secure or food insecure?*
- *Are there aspects of food security that are specific to being Indigenous?*
  - *Are there inter-generational effects of food insecurity?*
  - *Are cultural or traditional foods related to food security?*
- *In recent years there has been scholarship to redirect discussions of food systems to discussions on what it means to be part of a relational and kin-centric foodway. Does food security mean something different in the context of a relational or kin-centric foodway?*
- *I wanted to share the USDA’s definition of food security and see what your thoughts are on it. (*shares slide with the following definition*)*
  - Food security is defined as: “access by all people at all times to enough food for an active, healthy life."
    - Food security includes at a minimum:
      - The ready availability of nutritionally adequate and safe foods.
      - Assured ability to acquire acceptable foods in socially acceptable ways (that is, without resorting to emergency food supplies, scavenging, stealing, or other coping strategies).
  - *Can you share any reactions you have to this definition and what you would change, add, or dispute in this definition?*
- **Share slide with USDA Food Security Screener**
  - *How does this measure differ from how you think food security should be measured?*
- *Before we end today, is there anything anyone would like to add?*
  - *Are there any questions you wish I would have asked or topics you wish we had covered?*
